# Effect of an Educational Program on Healthcare Professionals' Readiness to Support Patients with Asthma, Allergies, and Chronic Obstructive Lung Disease for Improved Medication Adherence

**DOI:** 10.1155/2020/1585067

**Published:** 2020-10-27

**Authors:** Malin Axelsson, Benita Björk, Ulrika Berg, Karin Persson

**Affiliations:** ^1^Malmö University, Faculty of Health and Society, Department of Care Science, Malmö, Sweden; ^2^The Knowledge Centre for Allergy, Asthma and COPD, Region Skåne, Skåne County, Sweden

## Abstract

**Purpose:**

The aim of this study was to strengthen the healthcare professionals' readiness to support patients who have asthma, an allergy, and COPD for better medication adherence.

**Methods:**

The design was an educational intervention in a study population (*n* = 70) consisting of 66 nurses and four other allied healthcare professionals working in primary care with patients diagnosed with asthma, allergy, or COPD in a county in southern Sweden. As part of two training days, an educational intervention—consisting of lectures and workshops—was conducted. Both qualitative and quantitative data were collected. The qualitative data were collected during the workshops when the participants worked with fictitious patient cases. They documented in writing how they, based on the theoretical content in the educational intervention in combination with their clinical experiences, reasoned that the fictitious patients could be supported for better adherence. This documentation constituted qualitative data. The quantitative data were collected through questionnaires, which the participants completed before and after the intervention. Data from the questionnaires were statistically analyzed using descriptive statistics and paired *t*-tests. The qualitative data collected from the workshops were analyzed with content analysis.

**Results:**

The intervention increased the participants' knowledge of adherence (pre mean 3.95 versus post mean 4.18, *p*=0.001) and how to better support patients' adherence to medication (pre mean 3.71 versus post mean 3.98, *p*=0.001). Moreover, their knowledge of how to measure patients' adherence behavior (pre mean 3.02 versus post mean 3.54, *p*=0.001) and how to communicate with patients effectively about adherence was heightened (pre mean 3.92 versus post mean 4.13, *p*=0.011). Furthermore, participants felt that their readiness to support patients for better adherence had strengthened (pre mean 3.78 versus post mean 4.13, *p*=0.001). Individual adherence support for three fictitious patients with different adherence issues was developed.

**Conclusion:**

An educational intervention focusing on adherence and communication equipped healthcare professionals with tools to support patients with asthma, an allergy, or COPD for better medication adherence.

## 1. Introduction

A plethora of studies shows that medication adherence among individuals with asthma or chronic obstructive pulmonary disease (COPD) needs to be improved [1–7]. Low adherence to medication can compromise the chances of achieving good disease control and good health-related quality of life [8, 9]. Consequently, low medication adherence for asthma and COPD is associated with increased healthcare utilization and increased healthcare costs [10, 11]. Thanks to the extensive research on adherence in recent decades, several factors that affect patients' adherence behavior to medication treatment for asthma and COPD have been identified. Some factors can be attributed to the individual, such as personality traits [12–17]. Persons with low levels of the personality traits of conscientiousness or agreeableness [13, 15, 16] or high levels of neuroticism are more likely to report lower adherence to asthma medication treatment [12–17]. Illness perceptions in terms of being more concerned about the COPD [18] have been associated with lower adherence while perceptions that asthma can be controlled by treatment or understanding asthma have been associated with better medication adherence [19]. Beliefs about medication have also been associated with adherence among persons with asthma [13, 17, 19] and COPD [18, 20]. For instance, beliefs that the medication is a necessity for the health have been associated with better adherence among patients with asthma and COPD [13, 17, 19, 20] while concerns, i.e., worries about side effects or becoming addicted have been associated with lower adherence [13]. Indeed, both side effects and complex treatment regimens are of significance for adherence among patients with asthma and COPD [21]. Yet, another influencing factor of adherence can be health literacy [22, 23], which refers to a person's ability “to gain access to, understand, and use information” to promote and maintain health [24].

Another aspect of adherence is that healthcare professionals' adherence to treatment guidelines for asthma and COPD is low [25–28] which may jeopardize patients' adherence and disease management. Nonetheless, healthcare professionals who are treating patients with asthma and COPD have a responsibility to identify patients' potential poor adherence and to promote good patient adherence to medication treatment for these diseases [8, 9, 29]. This implies that patients' adherence to prescribed medication treatment for asthma and COPD should be checked at each follow-up visit at asthma/COPD clinics [8, 9]. Further, healthcare professionals should be well aware of factors influencing adherence [29, 30] and be knowledgeable about how to address adherence issues [29]. Nurses are a valuable resource for promoting patients' adherence [31] because they work closely with patients and possess the necessary competence to support improved medication adherence, for instance, by monitoring inhaler technique and communicating with patients about their adherence behavior [32]. In Sweden, as in many countries [29], most patients with asthma and COPD are treated at asthma/COPD clinics in primary healthcare centers; these clinics are usually led by nurses highly educated in the care and treatment of asthma, allergies, and COPD and by physicians with competence in the field. In addition, nurses with competence in asthma, allergy, and COPD care independently follow up prescribed medication treatment [33]; this may also include medication adherence. However, a recent study conducted in Sweden showed that poor asthma control and emergency visits are common and that these outcomes were attributed to irregular use of maintenance medication treatment, which was used as a proxy for poor adherence. It was concluded that there is a need for interventions to improve the management of asthma in primary care [34]. It has been argued that healthcare professionals working in primary care, where most asthma and COPD patients are treated, play an important role in improving adherence. Moreover, interventions aimed at improving adherence that healthcare professionals in primary care can implement in their daily clinical practice reach many patients with asthma and COPD. [29] Importantly, previous research shows that that asthma/COPD nurses sometimes feel uncertain when educating patients [35] and that an educational intervention among physicians increased their readiness to communicate about aspects of asthma with the patients [36]. It is, therefore, of importance to develop tools to follow if educational interventions increase health professionals' readiness to communicate about adherence with patients with both COPD and asthma. Consequently, an educational intervention with a focus on adherence and professional communication was carried out for nurses and other healthcare professionals working at asthma/COPD clinics in primary healthcare centers. The aim was to strengthen their readiness to support patients who have asthma, an allergy, and COPD for better medication adherence.

## 2. Materials and Methods

The study design was an educational intervention, during which both quantitative and qualitative data were gathered and subjected to analyses. This was a collaborative project between Malmö University in Sweden and The Knowledge Centre for Allergy, Asthma, and COPD, Region Skåne, Sweden.

### 2.1. Study Population

The study population consisted of asthma, allergy, and COPD nurses and other allied healthcare professionals working in primary care in a county in southern Sweden. The following inclusion criteria were used: being a registered nurse or an allied healthcare professional working at asthma/COPD clinic and participating during two annual training days. These annual training days are directed toward all registered nurses and allied healthcare professionals working at asthma/COPD clinics in primary care in one county in Sweden. The sample in the current study comprised those that participated during the training days.

### 2.2. Intervention

**Figure 1:**
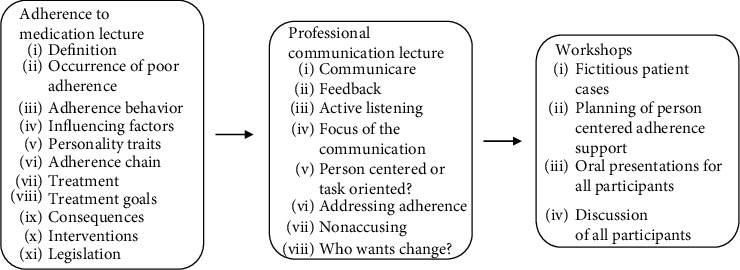
Overview of the content in the educational intervention.

An educational intervention, lasting 3½ hours, was carried out on two training days. The content in the intervention was based on the research team's overall and extensive competencies within this specific area. This means that the educational intervention was based on evidence, scientific literature on adherence, and proven experience within the research group altogether, which have been gained through adherence research, clinical practice within the field of asthma, allergy, and COPD, experience from education, planning of education, and content of lectures both at universities and in clinical practice. All of them were relevant for planning an educational intervention focusing on adherence. The intervention, which is depicted in detail in Figure 1, consisted of an educational package focusing on medication adherence and professional communication focusing on adherence. A lecture focusing on adherence to medication treatment was given, followed by a lecture focusing on professional communication. After these lectures, the research group performed three different fictitious patient cases with different adherence issues developed from clinical experiences within the Knowledge Centre. The patient cases are described in brief in Table 1. Thereafter, the healthcare professionals participated in one workshop each where they discussed different ways to support the three patients for better adherence according to patient-centered care. In a follow-up, each group orally shared their experiences of how to support better adherence and then engaged in a group discussion.

### 2.3. Data Collection

The data were collected in April 2018. Both quantitative and qualitative data were gathered to answer the aim. The quantitative data consisted of self-reported background data and self-reported readiness to support adherence. First, the participants completed questionnaires on background data. Then, they estimated their readiness to support adherence, which was done both before and after the educational intervention. The questionnaire included thirteen statements that were answered on a five-graded Likert scale, ranging from disagree = 1, do not agree = 2, neither or = 3, agree = 4, to agree completely = 5. To the best of our knowledge, there were no published questionnaires measuring healthcare professionals' readiness to support adherence; therefore, the questionnaire used in the current study was developed for that purpose, and this study is the first step to test the reliability of this questionnaire. The statements in the questionnaire were developed to correspond with the content in the educational intervention, which in turn was developed based on the competencies within the research team, i.e., adherence research, clinical expertise, teaching experiences, and relevant literature. First, two of the researchers developed a set of statements for the questionnaire. Thereafter, the other two researchers checked the relevance of the statements. This was done to test the face validity of the questionnaire [38]. The qualitative data were collected during the workshops through the participants documenting in writing how they, based on the theoretical content in the lectures and their clinical experiences, reasoned that the fictitious patients could be supported for better adherence; this documentation constituted the qualitative data.

### 2.4. Analysis

Background data and data collected through the questionnaires were analyzed with descriptive statistics (frequencies, percentages, means, and standard deviations). Paired *t*-tests were used to evaluate the intervention regarding readiness to support patients for better adherence. The qualitative data collected from the workshops were analyzed with content analysis. The data were compiled into three data sets, one for each case representing results from all the groups (*n* = 11) participating in the workshops. The three data sets contained ideas of how the three different cases were to be managed to improve adherence. In the analyses, the ideas for each case were merged as a narrative [39].

### 2.5. Ethical Considerations

The study adhered to the Helsinki Declaration [40] and to the directives of Malmö University, both of which are in line with Swedish legislation on research ethics [41]. No personal or sensitive data were gathered from the participating healthcare professionals. The participants received information in writing about the study when registering for the training days, and the information was followed up orally during the training days. They were informed that participation was voluntary and that they could withdraw at any time without explanation. The participants consented to participate in the study by choosing to fill in the questionnaires and by submitting written ideas and suggestions during the workshops. Furthermore, they were informed that participation in the study was completely independent of participation in the intervention (educational package focusing on adherence), that the collected data from the questionnaires and workshops would be handled confidentially, and that the results would be presented at the group level, so no individual could be identified. The questionnaires were completed anonymously without any codes that could be linked to any participant.

## 3. Results

**Figure 2:**
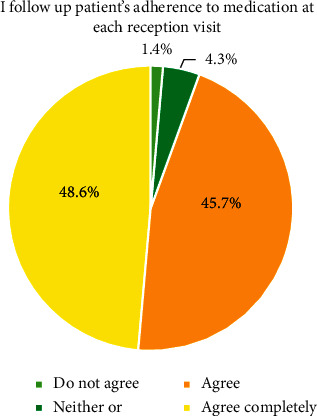
Proportions of healthcare professionals who follow up adherence to medication.

The results are based on 70 participants of which a majority were nurses (94.3%). A total of 84.3% of the participants had undergone education in the field of asthma, allergy, and COPD, and a half (55.1%) had worked at a certified asthma/COPD clinic in primary healthcare (Table 2). Certification of asthma/COPD clinic means that the primary healthcare center can guarantee that the care of patients with asthma/allergy/COPD is quality-assured and provided by qualified healthcare professionals in accordance with the recommendations of The National Board of Health and Welfare [42]. The average work experience within the field of primary healthcare was 7.0 years. As illustrated in Figure 2, 48.6% completely agreed and 45.7% agreed with the statement “I follow up patients' adherence to the medication at each reception visit.” The majority disagreed with the following three statements: “It is not part of my duties to follow up how the patients take their medication (91.4%), “Patients' adherence to medication need not be followed up at each reception visit” (77.1%), and “The patient's inhalation technique need not be checked at each reception visit” (82.6%).

### 3.1. Preintervention and Postintervention

As presented in Table 3, the intervention increased readiness to support patients to better adherence to some extent. After the intervention, participants scored significantly higher regarding their knowledge of adherence to medication and how to support patients for better adherence. Moreover, they reported higher readiness regarding how to ask patients about their adherence behavior, how to measure patients´ adherence behavior, and how to create an effective conversation with the patients. Further, they also disclosed increased readiness to support patients for better adherence to medication. However, there were no differences between pre- and postintervention regarding what extent they found it easy to talk about adherence, knowledge of what factors influence medication adherence, and the risks of poor adherence.

**Figure 3:**
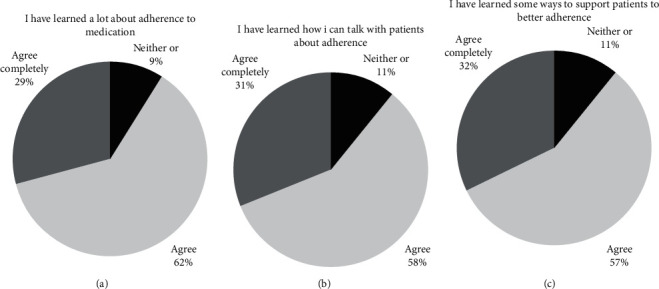
Presenting the participants' self-reported learning outcome of the intervention.

Figures 3(a)–3(c) show that the majority of participants stated that they had increased their readiness by having learned about adherence, having learned how to talk about adherence, and having learned how to support patients with asthma, allergy, or COPD for better adherence.

### 3.2. Results from the Workshops

#### 3.2.1. Patient Number 1

During the workshops, the participants discussed how to best support patient number 1, who displayed both skepticism and reluctance, for better adherence (Table 1). They suggested that initial communication should concern ascertaining what she knows about asthma and the medications and what she wants to know. Should she seek information about asthma, it was suggested that the verbal information be combined with pictures to facilitate her understanding of the anatomy and pathophysiology of the airways of an asthmatic. One could, for example, draw a picture or use teaching material denoting both healthy and obstructive airways. In addition, it was recommended that the score of the asthma control test [43] be used when talking about asthma symptoms and everyday consequences for this patient. To facilitate her understanding, examples were given on how the medication worked and what happens if the medication was not used. Because this patient had concerns about using the corticosteroid inhalation and because she did not adhere to the prescribed medication, the participants related the importance of explaining to her why medications are crucial and why inhaling corticosteroids are less dangerous than having swollen/inflamed airways. Here, they suggested that pictures of both healthy and obstructive airways be used to explain the effect of the corticosteroids. Another suggestion to address this patient's concerns with the medication was to draw parallels with other medications, for instance, the use of paracetamol when needed in the same way as one uses short-acting-beta-2-agonist as a reliever when having asthma symptoms.

The participants suggested that the self-care should focus on the patient's own goals: they suggested to include follow-ups, both at the asthma/COPD clinic in the primary healthcare center and through telephone calls. Further, they recommended a written self-care plan and a peak-expiratory flow meter to enable the patient to document and follow improvements and deteriorations with and without asthma medication.

#### 3.2.2. Patient Number 2

During the workshops, the participants discussed how to best support patient number 2 (Table 1) for improved adherence to asthma medication. They conveyed the importance of respecting the patient's own opinions while trying to initiate traditional treatment. They wanted to determine the reasons behind her skepticism and, perhaps, fear of inhaled corticosteroids. The participants suggested earning her trust through being patient and having a conscientious initiation and long-term planning. Furthermore, they suggested that this patient be informed about anatomy and pathophysiology with the help of pictures or drawings. An example was the use of Internet information videos as a means to explain her symptoms as coming from her lungs, not her heart.

Participants advised drawing spirometry curves to show the differences between taking medications and not. Moreover, it was proposed that patient number 2 needed repeated information and education concerning how asthma medications work preventively: short-acting with a long-term effect. This could be realized through, for instance, emphasizing that regular use of cortisone inhalations helps to maintain normal lung function and prevent chest tightness. Participants communicated that they would try to get this patient to give the medication a chance. Thus, they suggested that she started with short-acting-beta2-agonists, thereby enabling her to feel the immediate effect of the medication. In addition, they proposed she be given a written treatment plan. They underlined the importance of letting patient number 2 choose the inhaler and of then informing her how it worked. To facilitate this, both additional and repeated information about how the inhaler worked would be given.

Regarding self-care for patient number 2, it was suggested that she might benefit from a patient-training group, i.e., an asthma school for peer-support. Participants also discussed whether she might need some kind of psychological counseling, due to her anxiety, and future support in the form of exercise or yoga/mindfulness. Moreover, there was concern that her asthma might be exacerbated through her occupational use of hairdressing products. However, the priority was to motivate her to return to the asthma/COPD clinic in the primary healthcare center.

#### 3.2.3. Patient Number 3

To improve patient number 3s (Table 1) adherence, the participants recommended support with the practical inhalation technique, including loading of the medication. Moreover, he should have a written treatment plan. It was advised that the asthma/COPD nurse be contacted when new inhalers were prescribed so instructions on use should be given. If problems with the correct inhaler technique, spray + spacer, were discussed, he could, as an alternative, during follow-ups receive information about the new medication, which could be evaluated, for instance, by using the COPD Assessment Test (CAT™) [44]. However, participants also recommended a timely consultation with an asthma/COPD nurse, preferably in the presence of a next-of-kin, as patient information and education were considered important for this patient. He was deemed to need more information regarding COPD, learning early signs of exacerbation, and knowing how to avoid ending up in emergency care. For COPD, they recommended displaying uncomplicated anatomy with posters of healthy airways. Though patient number 3 should be praised for smoking cessation, it was considered important that he received information about oxygen uptake. Because he worked as a welder, the participants considered it important to remind patient number 3 of his work environment and the importance of using respiratory protection. Future planning for patient number 3 was to encourage him to initiate contact with a patient association for peer-support, to initiate training for regular exercise, and to invite him to participate in COPD school, i.e., patient-adapted education. In the future, participants needed to plan for interprofessional care and treatment.

## 4. Discussion

The aim of the current study was to strengthen the healthcare professionals' readiness to support patients who have asthma, an allergy, and COPD for better medication adherence. The results showed that the majority of the participating healthcare professionals checked patients' adherence at each visit. Furthermore, they saw it as their duty to check for adherence when patients visited the asthma/COPD clinic. However, it would have been preferable that all had measured adherence at each visit in order to comply with international guidelines [8, 9]. Comparisons between preintervention and postintervention revealed that the educational package seemed to have strengthened the healthcare professionals' readiness to support patients with asthma, an allergy, and COPD for better medication adherence. The workshops resulted in individual adherence planning for the three fictitious patients that was in line with both international [8, 9] and national guidelines [42].

As nurses have a unique combination of competence as caregivers and as patient educators, they play a significant role in supporting patients with asthma [45], allergies [46], and COPD [47] with the management of their health conditions. A systematic review shows that educational interventions conducted by nurses supported patients with asthma in acquiring more knowledge of the condition. This helped them to better manage their asthma, which in turn can contribute to a reduction of asthma morbidity and symptoms [48]. However, it has been reported that asthma/COPD nurses sometimes feel uncertain when educating patients [35]. Therefore, an intervention in the form of an educational package consisting of lectures and workshops aimed at heightening the readiness of healthcare professionals, the majority of them being nurses, to support patients for better medication adherence for asthma, allergies, and COPD was considered prudent. Raising their readiness may contribute to them supporting their patients' improved medication adherence. The intervention not only increased the participants' knowledge of adherence but also their knowledge about how to support patients for better adherence and how to create an effective conversation about adherence. Additionally, their readiness to support patients for better medication improved. Our findings are in line with a former study based on physicians that showed that intervention increased readiness to communicate about aspects of asthma, such as symptoms and treatment goals, after the participants had an educational intervention [36]. From an educational viewpoint, the current study shows that an adherence intervention increases nurses' and other healthcare professionals' awareness of adherence issues among patients with asthma and COPD and their readiness to address these issues. From a clinical viewpoint, this implies that educational interventions focusing on adherence similar to the one presented in this study targeting nurses and other healthcare professionals can be offered regularly. Thereby, nurses and other healthcare professionals become empowered enough to empower the patients to better adherence. As adherence to medications for asthma and COPD continues to be low [1–7] and leads to increasing healthcare costs [8, 9] and poor health outcomes [10, 11], addressing adherence is a continuous work and empowering patients to better adherence requires know-how. Thus, in order to be able to tailor the educational readiness of health care professionals, it is of importance to monitor which interventions increase the interest of talking about adherence with their patients. Medication adherence is a complex phenomenon, which is influenced by a number of different factors. Those are related to the patient, the disease and associated treatment, the healthcare system, and healthcare professionals [49]. Together, this argues that improving adherence requires joint efforts in cooperation between different factors such as patients, healthcare professionals, healthcare providers, and policymakers and is not an exclusive responsibility for patients, nurses, and other healthcare professionals.

Although a former study showed that patients with asthma who regularly attended asthma follow-up consultations in primary health care had better adherence [2], there remains considerable room for improvement concerning medication adherence for both asthma and COPD [1–7]. Most likely, tailored interventions are needed if adherence is to be improved, as one method does not fit all. This places demands on healthcare professionals who need knowledge about how adherence support can be tailored to each patient [21]. To practice developing individual adherence support, the participants in the current study were presented for three fictitious patients who all three had different reasons for nonadherence to the prescribed treatment. During the workshops, the participants practiced developing tailored interventions to act on the patients' poor adherence. One of the fictitious patients, patient number 1, was reluctant to accept her asthma diagnosis; consequently, she was hesitant to use the prescribed asthma medication. Poor adherence to asthma medication treatment can be seen as a rational response from a patient perspective. Questioning one's diagnosis or believing that asthma is not an acute condition is perceptions of illness, which have been associated with adherence to asthma medication treatment [19, 50]. During the workshops, the healthcare professionals, for instance, suggested addressing patient number 1's illness perceptions by focusing on what she knows about asthma and the medications and on what she wants to know. Both patient number 1 and number 2 held concerns about their asthma medication and corticosteroids, in particular. Assumptions about medications are recognized as influencing factors of adherence. Patients with asthma who have concerns about the medication are less likely to be adherent in comparison to those who regard the medication as necessary for their health [13]. Moreover, because patients with asthma who are followed up regularly at asthma/COPD clinics in primary health centers are more inclined to see their medication as necessary, this may indicate that healthcare professionals can influence patients' assumptions [2]. During the workshops, the participants suggested that the concerns about the medication that both patient number 1 and number 2 had needed to be addressed. Importantly, they proposed different approaches. For patient number 1, a more educational approach was planned, while for patient number 2, the importance of them being conscientious and having a long-term plan to win her trust was underlined. Personality characteristics are associated with adherence behavior. For instance, worriers like patient number 2 are more likely to be less adherent to asthma medication treatment [13–16]. Thus, healthcare should be aware of patients' different personality characteristics when planning for adherence support as individual differences influence adherence behavior. Previous research has shown that the personality traits of agreeableness, conscientiousness, and neuroticism are associated with adherence to asthma medication [12–17]. Persons with lower levels of agreeableness tend to be skeptical and less into cooperation, persons with lower levels of conscientiousness are less goal-directed, and persons with higher levels of neuroticism are more worried and anxious [51]. These personality characteristics are by nature associated with lower adherence [13]. Increased awareness of how personality characteristics influence adherence is important in efforts to promote adherence and, therefore, was a necessary ingredient in the current educational interventions aimed at heightening healthcare professionals' readiness to support patients to better adherence. During the current workshops, the participants practiced planning tailored adherence support for patient number 1, who was reluctant and skeptical about her asthma diagnosis, and for patient number 2 who was worried and anxious.

Nurses play a central role in educating patients about practical inhaler techniques [32]. Indeed, nurse-led education has been successful in improving the inhaler technique for patients with asthma [52] and with COPD [53]. In the current study, the fictitious patient number 3 who had COPD was unintentionally nonadherent—meaning that he expressed a willingness to adhere to the prescribed medication for COPD; however, adherence failed due to incorrect inhaler technique [21]. During the workshops, the planned adherence support for this patient included a written treatment plan stating, for instance, that the asthma/COPD nurse should be contacted when new inhalers were prescribed. The nurse could then instruct patient number 3 on how to use the new ones; this would serve to minimize the risk for unintentional nonadherence. Because poor inhaler technique is common among patients with COPD [54] and asthma [55], a suggestion for a clinical implication is that educational interventions to teach patients with COPD and asthma correct inhaler technique are routinely introduced as a health policy for asthma/COPD clinics. Additionally, timely consultation with an asthma/COPD nurse, preferably in the presence of a next-of-kin, was planned for patient number 3 because he seemed to be in need of more information and education about COPD and the treatment. This may indicate that this patient had lower health literacy, which is an essential influencing factor of adherence among patients with COPD [23]. Therefore, it is of vital importance to check that the patient understands the health information provided.

### 4.1. Methodological Considerations

A strength of the current study was the sample and their competence within the field. The participants had several years of clinical work experience at asthma/COPD clinics in primary healthcare centers. Additionally, the majority had undergone theoretical education within the field of asthma, allergy, and COPD care. In Sweden, it is mandatory to have theoretical education in this field to work at asthma/COPD clinics, which may not be the same in other countries. This potential difference in education between countries could, thus, interfere with the representativeness of the sample. Despite their competence, the intervention increased their readiness to support patient adherence. This increase may be ascribed to the possibility of discussing and sharing clinical experiences with colleagues from other clinics. Further, the discussion and sharing of clinical experience took part in the content in the educational intervention. This may indicate that education about adherence is to be included in syllabi in both theoretical and practical education for healthcare professionals and be continued thereafter. Regarding the generalizability of the results, the clinical work experience and theoretical education among the participants need to be taken into consideration. If the participants had had less experience, maybe, the effect of the intervention and the content in the adherence support generated from the workshops had been different. Most of the sample consisted of nurses, which could be considered a strength as both nurses and physicians lead the asthma/COPD clinics in primary health care. At the same time, not having other healthcare professionals attend the training days can be seen as a limitation, as a treatment for asthma and COPD involves interprofessional competence. When designing the study, there was no questionnaire available for measuring healthcare professionals' readiness to support patients to better adherence. Therefore, a questionnaire was developed for that purpose, and it may serve as an implication for future research focusing on further development and testing for the questionnaire measuring healthcare professionals' readiness to support patient adherence. Importantly, the statements in the questionnaire were based on the competencies within the research team, which included experience from individual adherence research, clinical expertise within the field, and teaching skills with a special focus on communication. To test face validity [38], the statements in the questionnaire were developed by two of the researchers and the other two checked the relevance of the statements for measuring readiness for adherence. This overall competence argues that the content in both the educational intervention and the questionnaire is valid for its purpose: to raise healthcare professionals' readiness to support patients to better adherence and to measure readiness. As to reliability, the questionnaire was used in two different educational sessions, and the results from both groups were comparable, indicating that the next step could be to do a test-retest reliability check of the questionnaire. Moreover, the study design did not enable an evaluation of the intervention with consideration to patients' adherence. However, improving communication behavior among physicians has resulted in better asthma outcomes [36]. Hopefully, the outcome of our study will inspire nurses and other healthcare professionals at asthma/COPD clinics to include adherence planning for patients, which in turn will improve adherence to medication treatment. Performing an educational intervention as in the current study but with one intervention group and one control group and evaluating the effect on patients' adherence is indeed an implication for future research.

## 5. Conclusion

An educational intervention consisting of an educational package of lectures and workshops increased healthcare professionals' readiness to support patients with asthma, an allergy, or COPD for better medication adherence. Workshops can be useful for practicing the planning of individual adherence support for patients with different adherence issues. This may indicate that educational interventions consisting of a combination of theoretical and practical content focusing on adherence equip healthcare professionals with tools to support patients with asthma, an allergy, or COPD for better medication adherence.

## Figures and Tables

**Table 1:** Description of the three fictitious patient cases.

*Patient number 1*: a 52-year-old woman diagnosed with asthma in childhood and prescribed regular corticosteroids for inhalation twice a day. She is very skeptical of both the asthma medication and the asthma diagnosis. She stops taking her asthma medication when she feels well. Presently, she has not taken it for the last four months. The medication was only used for two weeks after the last appointment at the asthma/COPD clinic. Thereafter, she felt well and stopped using the inhaler. In preparation for today´s appointment with the nurse at the asthma/COPD clinic at the primary healthcare center, she has taken the medication. The appointment is a follow-up, as the last spirometry showed an obstructive curve. The asthma control test, 18, which is a symptom scoring assessment, shows 15. This is to be interpreted as poor disease control, though she says she feels very well. She does not believe in asthma medication or the asthma diagnosis. Rather, she believes that there is a problem with her back, which a chiropractor, in fact, told her was the case

*Patient number 2*: a 52-year-old woman with a medical history of asthma and an allergy. Patient number 2 works as a hairdresser and loves jewelry and clothing. She is a worrier and very skeptical about and hesitant to use inhaled corticosteroids. As she believes that the body is self-healing, she prefers acupuncture to medication to get her body in balance. She is now visiting the nurse at the primary health care center because she has suffered from troublesome chest tightness and respiratory symptoms for the last six months; this has forced her to seek emergency care several times. She believes that there is something wrong with her heart, not with her respiratory airways. However, medical examinations show that her symptoms were not related to her heart but more likely to her airways. The patient is very worried and anxious. She does not believe she has asthma any longer because acupuncture healed her. For that reason, she has not used any asthma medication for years, and she has not shown up at the follow-up consultations at the asthma/COPD clinic at the primary healthcare center. Instead, she has sought health care for her heart problems

*Patient number 3*: a 63-year-old man, and a former smoker, diagnosed with COPD three years ago. He has worked as a welder for most of his working life. He has tried to use the available protective equipment as his workplace. He says he is fine. Patient number 3 was present at his father's death, which was a traumatic experience for him because his father had a very hard time breathing. Patient number 3 cannot cope as before, but he reasons that he is no longer young—63 years old. He cannot exercise but thinks it is enough with his work and some gardening. He has been prescribed short-acting-beta2-agonist and long-acting-antimuscarinic-antagonists (LAMAs) for inhalation use. When he refilled the prescription of the LAMAs, he received another inhalator. He was annoyed that he was prescribed a cheaper medication^∗^ without training about the correct inhalation technique, explaining that it was typical for the healthcare system to prescribe the cheapest one. Thereafter, his respiratory symptoms, mucus secretion, breathlessness, and fatigue increased. Eventually, he was hospitalized for COPD exacerbation. Now, he is visiting the primary healthcare center for a follow-up. He is provoked by the fact that he was prescribed a cheaper and low-quality medication stating, “They do not care about the patient; it's just about making money”. He is also questioning the value of visiting the primary healthcare center

^∗^Regarding the prescription of medication in Sweden, it is to be noted that there is a high-cost threshold as part of the Pharmaceutical Benefits Scheme. The threshold serves to protect persons from high costs for prescribed medication, and medications are subsidized via tax funds. Moreover, pharmacies are obliged to offer cheaper medication if available as the more expensive medication is not included in the high-cost threshold. However, inhaled drugs are one of the few medications that the pharmacy must not change without contacting the prescriber [37].

**Table 2:** Background characteristics of the study population (*n* = 70).

Occupation: registered nurses	*N* (%)
Asthma-COPD-allergy nurse	55 (78.6)
District nurse	4 (5.7)
Nurse	4 (5.7)
Nurse specialist	1 (1.4)
Research nurse	2 (2.9)

Other healthcare professionals	
Physiotherapist	3 (4.3)
Nurse assistant	1 (1.4)

Education	
Bachelor degree in nursing	
Yes	36 (53.7)
No	31 (46.3)
Bachelor degree in physiotherapy	
Yes	1
No	2
Master degree in nursing	
Yes	6 (8.7)
No	63 (91.3)
Education in asthma, allergy, COPD 15 ECTS	
Yes	59 (84.3)
No	11 (15.7)

Work experience	
Working at certified asthma-COPD reception	
Yes	38 (55.1)
No	31 (44.9)
Work experience in years	Mean (SD)
As a health care professional	19.5 (12.2)
At asthma, allergy, COPD reception	7.0 (5.8)

COPD = chronic obstructive pulmonary disease; ECTS = European Credit Transfer and Accumulation System; SD = standard deviation.

**Table 3:** Comparisons between preintervention and postintervention regarding readiness for adherence support.

Variable	Mean (SD) Preintervention	Mean (SD) Postintervention	*p* value^##^
I believe I have good knowledge of adherence to medications	3.95 (0.65)	4.18 (0.53)	0.001
It's easy to talk about adherence	3.82 (0.81)	3.88 (0.78)	0.484
I have good knowledge of how I can support patients for better adherence	3.71 (0.76)	3.98 (0.62)	0.001
It is important to know which factors influence medication adherence	4.46 (0.79)	4.54 (0.69)	0.496
I know how to ask patients about their adherence behavior	3.68 (0.79)	3.95 (0.60)	0.007
I know the risks of poor adherence	4.29 (0.76)	4.38 (0.55)	0.292
I know how to measure patients' adherence behavior	3.02 (1.05)	3.54 (0.96)	0.001
I know how to create an effective conversation with the patients	3.92 (0.63)	4.13 (0.58)	0.011
I feel that I have the readiness to support patients for better adherence to medication	3.78 (0 .72)	4.13 (0.65)	0.001

SD = standard deviation. ^##^Paired samples *t*-test.

## Data Availability

The data used to support the findings of this study have not been made available because of legal reasons.
